# Lipidomic Phenotyping Reveals Extensive Lipid Remodeling during Adipogenesis in Human Adipocytes

**DOI:** 10.3390/metabo10060217

**Published:** 2020-05-26

**Authors:** Florian Miehle, Gabriele Möller, Alexander Cecil, Jutta Lintelmann, Martin Wabitsch, Janina Tokarz, Jerzy Adamski, Mark Haid

**Affiliations:** 1 Research Unit Molecular Endocrinology and Metabolism, Helmholtz Zentrum München, German Research Center for Environmental Health GmbH, 85764 Neuherberg, Germany; florian.miehle@helmholtz-muenchen.de (F.M.); gabriele.moeller@helmholtz-muenchen.de (G.M.); alexander.cecil@helmholtz-muenchen.de (A.C.); lintelmann@helmholtz-muenchen.de (J.L.); janina.tokarz@helmholtz-muenchen.de (J.T.); adamski@helmholtz-muenchen.de (J.A.); 2 German Center for Diabetes Research (DZD), 85764 Neuherberg, Germany; 3 Division of Pediatric Endocrinology and Diabetes, Department of Pediatrics and Adolescent Medicine, University Medical Center Ulm, 89075 Ulm, Germany; martin.wabitsch@uniklinik-ulm.de; 4 Lehrstuhl für Experimentelle Genetik, Technische Universität München, Freising-Weihenstephan, 85764 Neuherberg, Germany; 5 Department of Biochemistry, Yong Loo Lin School of Medicine, National University of Singapore, Singapore 117596, Singapore

**Keywords:** adipocytes, adipogenesis, differential mobility spectrometry (DMS), lipidomics, lipidyzer, mass spectrometry, metabolomics, phenotyping, Simpson-Golabi-Behmel syndrome (SGBS)

## Abstract

Differentiation of preadipocytes into mature adipocytes is a highly complex cellular process. At lipidome level, the adipogenesis remains poorly characterized. To investigate the lipidomic changes during human adipogenesis, we used the Lipidyzer^TM^ assay, which quantified 743 lipid species from 11 classes. The undifferentiated human SGBS cell strain showed a heterogeneous lipid class composition with the most abundant classes, phosphatidylethanolamines (PE), phosphatidylcholines (PC), and sphingomyelins (SM). The differentiation process was accompanied by increased ceramide concentrations. After completion of differentiation around day 4, massive lipid remodeling occurred during maturation, characterized by substantial synthesis of diacylglycerols (DAG), lysophosphatidylethanolamines (LPE), PC, PE, SM, and triacylglycerols (TAG). Lipid species composition became more homogeneous during differentiation to highly concentrated saturated and monounsaturated long-chain fatty acids (LCFA), with the four most abundant being C16:0, C16:1, C18:0, and C18:1. Simultaneously, the amount of polyunsaturated and very long-chain fatty acids (VLCFA) markedly decreased. High negative correlation coefficients between PE and PC species containing VLCFA and TAG species as well as between ceramides and SM imply that PE, PC, and ceramides might have served as additional sources for TAG and SM synthesis, respectively. These results highlight the enormous remodeling at the lipid level over several lipid classes during adipogenesis.

## 1. Introduction

Overweight and obesity have increased dramatically in recent decades and now affect hundreds of millions of people worldwide, reaching pandemic levels [1]. Obesity has an adverse impact on a range of physiological processes, thereby increasing the risk of developing diseases like type 2 diabetes [2], cardiovascular diseases [2,3], and some types of cancer [4]. Overweight and obesity are mostly characterized by an excess of white adipose tissue (WAT). Adipocytes, the main constituent of this tissue, control the energy balance by storing triacylglycerols in periods of energy excess and breaking down these lipids during energy deprivation. However, the physiological role of adipocytes is much more complex than simply acting in energy storage. These cells secrete numerous diverse lipids and proteins controlling and regulating various bodily functions like appetite, immunological and inflammatory responses, and blood pressure and thereby act as an endocrine organ [5,6].

The development of adipocytes from precursor cells is known as adipogenesis. Within this differentiation process, fibroblast-like preadipocytes differentiate into lipid-laden and insulin-responsive adipocytes. This highly complex process involves the concerted interaction of a cascade of transcription factors like peroxisome proliferator-activated receptor gamma (PPARγ) and CCAAT/enhancer-binding proteins (C/EBPs) as well as different metabolic pathways including the TCA cycle, fatty acid synthesis, glycolysis, and polyamine biosynthesis [7,8,9]. The differentiation process has been characterized predominantly in murine cells, using different omics approaches like transcriptomics [10,11], metabolomics [9,12], proteomics [13,14,15], as well as the combination of transcriptomics and metabolomics [16]. However, lipids and their precise composition changes were so far sparsely characterized due to a lack of appropriate “high-resolution” mass spectrometry (MS) methods and internal standards. Several years ago, Roberts and coworkers characterized the levels of free fatty acids as well as the total levels of fatty acids of triacylglycerols (TAG) and glycerophospholipids in differentiating murine 3T3-L1 cells [9]. Two other studies analyzed the levels of some phosphatidylcholines (PC) using the same cell model, but without resolving the lipid isobars in experimental setups that were focused on the analysis of polar analytes [16,17]. Liaw and coworkers compared differentiated 3T3-L1 cells with primary mouse ear-derived mesenchymal stem cells and brown BAT-C1 adipocytes with a global lipid profiling method [18]. Additionally, they investigated the differentiation of murine adipocytes. However, they did not longitudinally analyze the whole adipogenesis process but compared only undifferentiated with fully differentiated 3T3-L1 cells. Even less knowledge on lipids has been compiled in the past for human adipocytes. Collins and coworkers analyzed the levels of fatty acid compositions of TAG and phospholipids without further class separation in primary adipocytes of subcutaneous origin [19]. To the best of our knowledge, the adipogenic process in a human cell model was so far not characterized with high-resolution lipidomics approaches. Therefore, we studied the development of human preadipocytes into mature adipocytes on a lipidomics scale with the recently developed Lipidyzer^TM^ (SCIEX, Darmstadt, Germany) method. We were able to simultaneously quantify 743 lipid species from 11 lipid classes. In combination with multivariate statistics, we could uncover correlations that suggest that extensive lipid remodeling occurs between several lipid classes during adipogenesis. These findings might contribute to the elucidation of new therapy strategies in obesity and other lipid metabolism affected disorders.

## 2. Results

### 2.1. Analytical Method Validation

In order to study the process of adipogenesis of human SGBS cells we decided to use the novel targeted Lipidyzer™ technology. Although originally developed and validated for the fast and automated analysis of human plasma samples [20], recently published studies also showed good performance of the Lipidyzer™ assay with other matrices than plasma [21,22,23,24,25]. With the aim to obtain a meaningful “fit for purpose method”, we investigated the analytical performance regarding linearity and repeatability for the SGBS cells.

For linearity evaluation, we used nine different volumes of cell homogenates (10, 20, 40, 50, 60, 80, 100, 200, and 300 µL) of undifferentiated and differentiated (day 15) SGBS cells and determined mean concentrations for the single lipid classes. For most classes, the coefficients of determination (R^2^) of the linear regressions were higher than 0.9 for both sample types, the differentiated and the undifferentiated cells, meaning we had good linearity of the method (Figure S1, Table S1). However, two classes, the dihydroceramides (DCER) and free fatty acids (FFA), revealed insufficient linearity for both sample types. The measurements of lactosylceramides (LCER) showed high linearity in the undifferentiated (*R*^2^ = 0.9734) but low linearity in the differentiated cells (*R*^2^ = 0.0479).

For repeatability evaluation, we calculated the relative standard deviations (CV) of the QC pooled samples. With the exception of DCER, all other lipid classes revealed CV values < 15% (Table S2). We also analyzed the background signals of the lipid classes by defining a lower cut-off value for the Lipidyzer™ method at a value of 1.5× the value measured in the blank. In total, 12 lipid classes were present in QC samples in quantities higher than 1.5× the blank values (Table S2). Only FFA showed up in lower apparent amounts because their measured concentrations were already high in blank samples. In conclusion, the Lipidyzer™ assay showed good linearity and repeatability for most of investigated lipids in SGBS cells.

Finally, we decided to exclude the FFA and DCER data from the data set due to their insufficient reliability in the validation testing. However, we left the LCER in the data set as we think that the drop of concentration between undifferentiated and differentiated cells is a noticeable result of our study.

### 2.2. Cellular Lipid Composition Undergoes Remodeling During Adipogenesis to Mainly TAG

Alterations in the lipid content of some lipid classes (e.g., TAG, phospholipids) in murine cells undergoing the process of adipogenesis have long been known [9,16,18,19]. However, concentration changes of many lipid species from multiple lipid classes at the different stages of human adipogenesis have not been investigated so far. We used the Lipidyzer^TM^ technology to follow the differentiation process of the human SGBS cell strain by quantifying the lipids of samples at days 0, 4, 8, 12, 16, and 20 of adipogenesis.

To track the successful cell differentiation of preadipocytes into lipid-laden adipocytes, we monitored the cellular process by microscopy (Figure S2) and analyzed relative mRNA levels of the main adipogenic transcription factors *PPARG* and *CEBPA* (Figure S3). Microscopic analysis showed enormous lipid storage in droplets starting between day 4 and 8. The analyses of the relative mRNA expression levels showed strong upregulation of *PPARG* (40.7 ± 9.5 fold change at day 12 compared to day 0) and *CEBPA* (52.0 ± 8.8 fold change at day 12 compared to day 0). These data demonstrate successful differentiation of SGBS cells into adipocytes.

**Figure 1:**
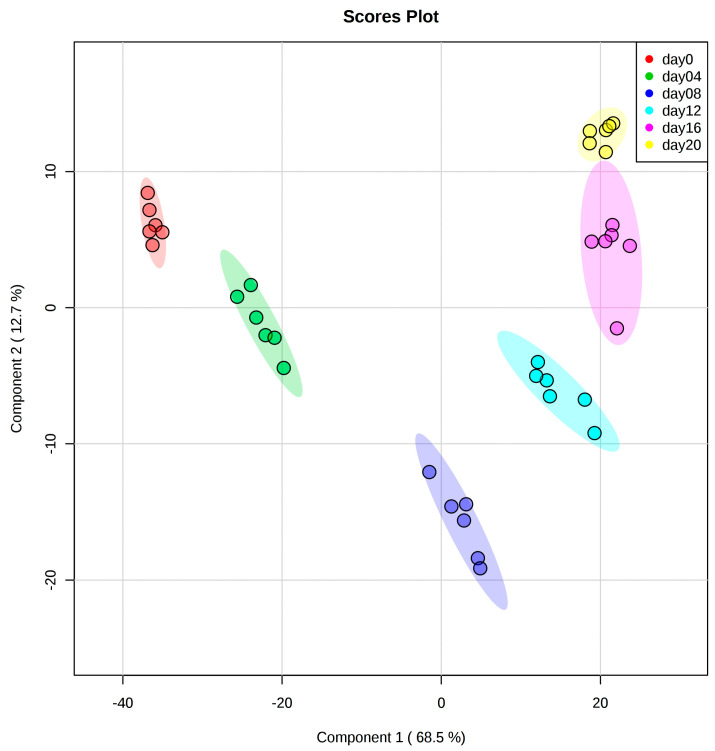
Partial least squares-discriminant analysis (PLS-DA) score plot showing very clear clustering of lipid species regarding the different time points of adipogenesis. While component 1 (68.5% variance) was sufficient to separate the early phase of differentiation (days 0, 4, and 8), the second component was necessary for separation of the later stages of differentiation (days 12, 16, and 20, 12.7% variance). The color code for the data points of the different days of adipogenesis is shown in the box inside the figure. Illustrated are also the 95% confidence intervals for each group. Cross-validation and permutation results confirmed the model to be predictive and not overfitted (R2: 0.97, Q2: 0.97; *p* < 5 × 10^−4^ (0/2000 permutation numbers), test statistics selected by separation distance (B/W)). Each group consisted of six samples.

We were able to simultaneously quantify 743 lipid species of 11 different lipid classes with the accurate identification of lipid isobars. To investigate putative differences between the lipid concentration levels at different time points, we conducted partial least squares-discriminant analysis (PLS-DA, Figure 1) and principal component analysis (PCA, Figure S4). PLS-DA shows a clear separation of the different time points of adipogenesis using the first two principal components with 68.5% and 12.7% of explained variance (Figure 1). While component 1 was sufficient to separate the early phase of SGBS differentiation (days 0, 4, and 8), the second component was necessary for separation of the later stages of differentiation (days 12, 16, and 20). Component 1 was mostly influenced by TAG species, whereas their influence on component 2 was lower (Table S3). 

**Figure 2:**
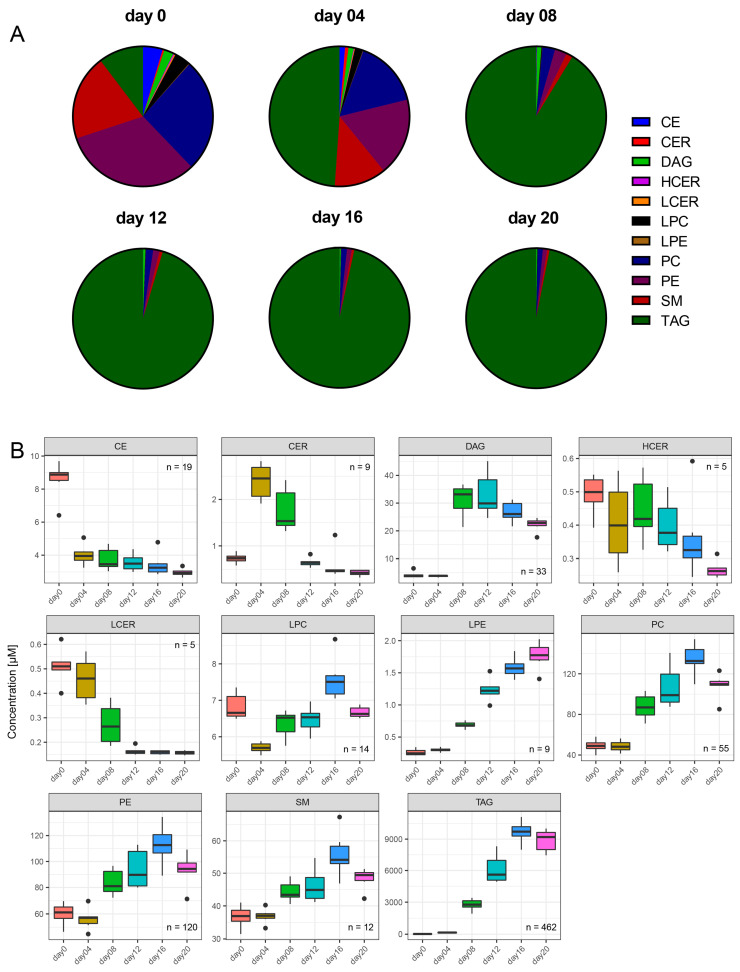
Changes in lipid class compositions and concentrations in specific lipid classes in the course of adipogenesis showed enormous lipid remodeling during the ongoing differentiation process. (**A**): Relative lipid class compositions at different days of adipogenesis in molarity %. Prior to differentiation at day 0, a very heterogeneous class distribution could be observed. This composition became more uniform during adipogenesis due to the predominance of triacylglycerols (TAG). The relative fraction for TAG increased from 10.3 ± 0.5 molarity % at day 0 to 96.9 ± 0.4 molarity % at day 20. (**B**): The concentration profiles of different analyzed lipid classes showed individual time courses during ongoing adipogenesis. Cholesteryl esters (CE), HCER, and LCER concentrations decreased strongly during adipogenesis, whereas LPE, PC, PE, and TAG concentrations strongly increased. Ceramide (CER) concentrations increased from day 0, peaking at day 4, and then decreased below the starting concentration levels. DAG species had their concentration maximum from days 8 to 12. LPC fluctuated during the whole differentiation process.

Next, we were interested in the lipid class compositions at the six time points of differentiation (Figure 2A). In preadipocytes, that is, at the start of differentiation (day 0), a very heterogeneous lipid composition was observed with the most dominant classes being the phosphatidylethanolamines (PE; 32.1% ± 0.9%), phosphatidylcholines (PC; 26.3% ± 0.4%), sphingomyelins (SM; 19.7% ± 0.6%), and TAG (10.3% ± 0.5%). While the relative proportions of PE, PC, and SM declined tremendously during the ongoing cell differentiation, the fraction of TAG increased from initially 10.3 ± 0.5% to finally 96.9 ± 0.4% at day 20. The relative fractions of all other lipid classes decreased strongly during the differentiation process to fraction sizes of finally 1.2% and lower. To conclude, the different stages of cell differentiation could be clearly distinguished based on the relative lipid compositions (Figure 2A) as well as by PLS-DA (Figure 1). Interestingly, the relative lipid class compositions did not reveal strong changes after day 8 of differentiation.

The time courses of the lipid species concentrations revealed an interesting alternative perspective on adipogenesis (Figure 2 B). Overall, we observed significant changes over time in the concentrations of 725 out of 743 lipids, that is, for 97.7% of all quantified lipid species. When looking at the lipid classes, only three of them, namely, CE (*p* = 4.52 × 10^−4^, Table S4), HCER (*p* = 5.23 × 10^−3^), and LCER (*p* = 2.80 × 10^−5^), showed continuous and significant decreases in concentration levels from day 0 to day 20 (Figure 2B). CER concentrations increased significantly from day 0 to day 4 of differentiation (*p* = 1.51 × 10^−5^) and subsequently decreased significantly below the limit of detection (LOD). In contrast, the TAG concentration levels increased continuously (*p* = 4.01 × 10^−6^). The TAG concentration levels were the highest among all classes and levels started from 19.2 ± 2.0 µM on day 0 to 8874.3 ± 1072.1 µM on day 20. LPE, PC, and PE concentrations also increased strongly during adipogenesis, whereas SM concentrations showed only a mild increase during differentiation (*p* = 5.04 × 10^−5^). In contrast, DAG concentrations increased from day 4 to day 8 and remained at a high level (*p* = 3.40 × 10^−5^). LPC concentrations fluctuated around the starting value during differentiation (*p* = 1.50 × 10^−4^).

### 2.3. The Most Abundant Fatty Acids in Differentiated Human SGBS Cells Are C16:0, C16:1, C18:0, and C18:1

**Figure 3:**
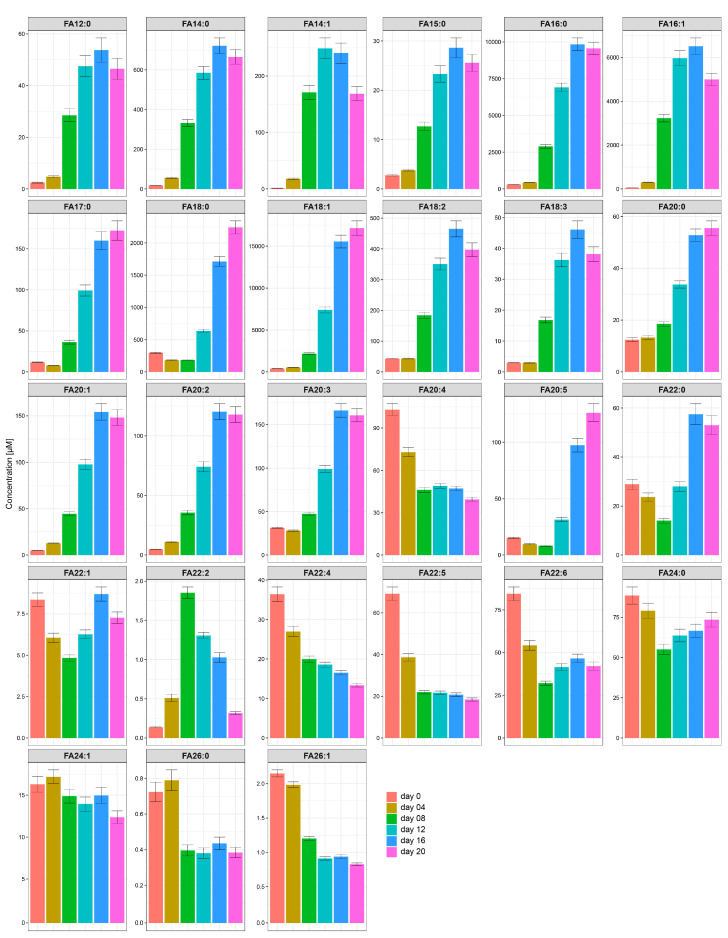
Time courses for the concentrations of the single FA side chains bound in the lipids summarized over all classes. Analysis of summarized lipids’ side chain concentrations over all classes revealed four dominant fatty acids, namely FA 18:1, FA 16:0, FA 16:1, and FA 18:0. Their concentrations increased strongly during adipogenesis together with the other LCFA (with the exception of FA 20:4) and the MCFA 12:0. In contrast, the very VLCFA mostly decreased during adipogenesis.

As we were interested in the concentration changes of the single fatty acid (FA) species during SGBS adipogenesis, we subsequently focused on a detailed analysis of the fatty acids bound to the lipid backbone. Figure 3 illustrates the time courses of the concentrations for the single FA side chains summarized over all lipid classes. With the exception of C20:4, all LCFA as well as the medium-chain FA (MCFA) lauric acid (C12:0) showed strong increases in concentration during adipogenesis. Among this group of FA, C18:1, C16:0 (palmitic acid), C16:1, and C18:0 (stearic acid)—in descending order—were the most abundant. In contrast to the MCFA and nearly all LCFA, most of the VLCFA decreased during adipogenesis. FA C22:0, C22:1, and C22:2 showed fluctuating concentration profiles.

**Figure 4:**
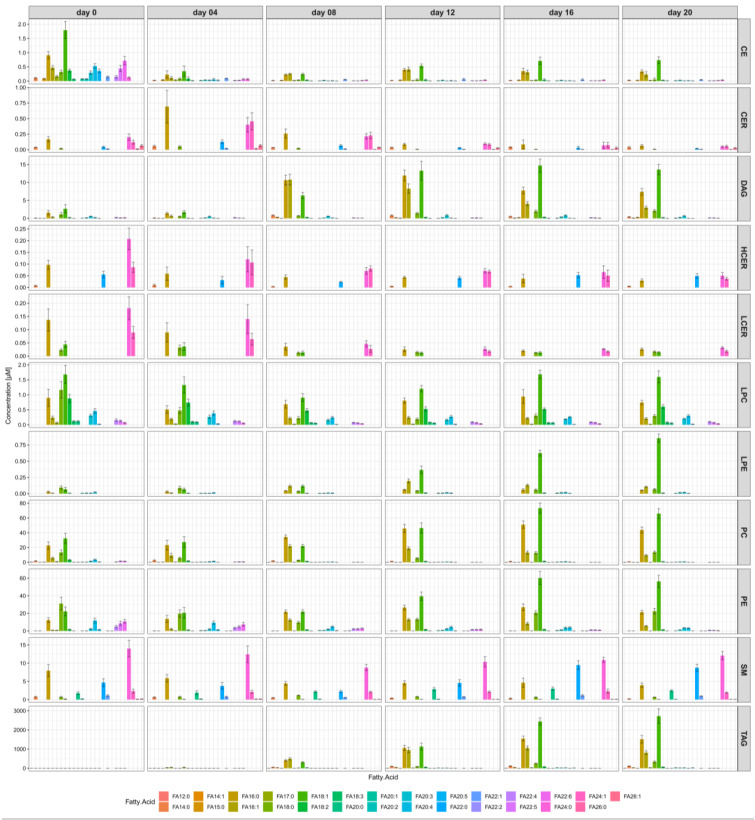
Fatty acid concentrations and compositions changed markedly during adipogenesis in all 11 lipid classes. At the start of differentiation, the lipids had a very heterogeneous side chain distribution with high concentration levels of LCFA and VLCFA. The concentrations of the VLCFA decreased during adipogenesis in all classes, with the exception of the class SM. The concentration courses of the LCFA were more complex because their levels increased markedly during adipogenesis within the classes DAG, LPE, PC, PE, and TAG, but decreased in the class CE. The FA concentration course of the SM differed strongly from the other classes because the VLCFA remained at high levels during adipogenesis.

A detailed analysis of the time courses of the concentrations for the single FA side chains separately for each lipid class allowed an even more detailed glimpse into adipogenesis (Figure 4). Illustrations on an enlarged scale can be found in Figure S5A–F. We were able to identify individual FA concentration changes over time of differentiation, which were strongly dependent on the lipid class.

The FA species of the three classes of ceramides, namely CER, HCER, and LCER, showed similar behavior in that the concentrations of the LCFA and VLCFA side chains decreased during adipogenesis. In contrast, the LCFA in DAG, LPE, PC, PE, and TAG substantially increased. In particular, the concentrations of C18:1 and C16:0 increased strongly during adipogenesis; for example, TAG-C16:0 increased from 2.5 µM at day 0 to 1515.1 µM at day 20. In contrast, VLCFA were present at only very low levels in the classes of DAG, LPE, PC, PE, and TAG. 

The FA composition of the SM differed strongly from the compositions of the other classes in that the SM comprised the highest absolute amounts of VLCFA. Especially C22:0 (behenic acid) and C24:0 (lignoceric acid) were present in SM at considerable concentrations. Their concentrations were increased up to a factor of 320 at day 20 compared with the other classes. The fatty acid concentrations of the LPC did not change markedly during adipogenesis. In contrast, the LCFA of the CE decreased to half the maximal concentrations and the VLCFA even more during the differentiation process.

### 2.4. Correlations between Concentration Profiles of Lipid Species from Different Lipid Classes Reveal Extensive Lipid Remodeling during Adipogenesis

**Figure 5:**
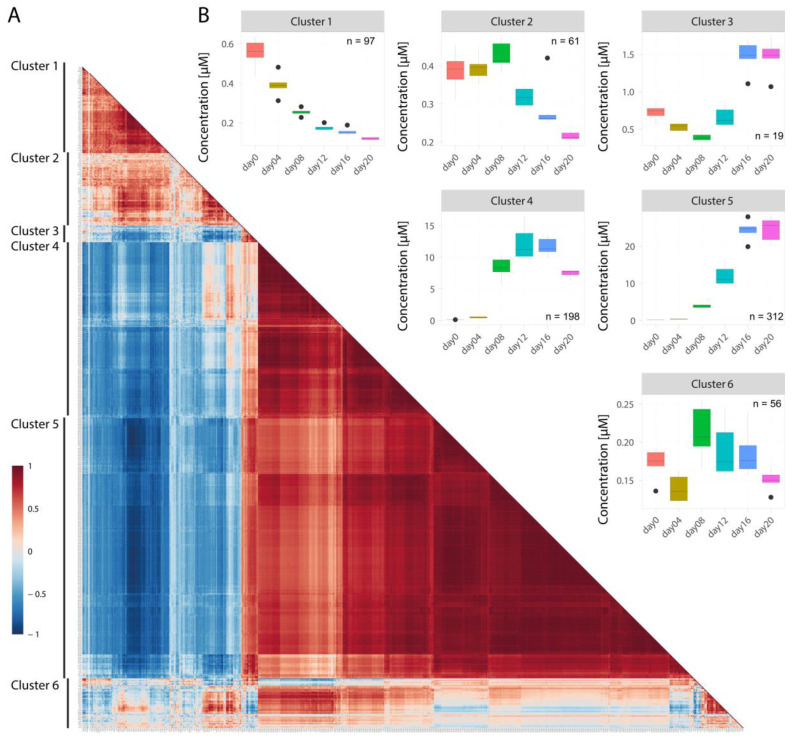
Spearman’s rank correlation analysis of lipid concentration trajectories during adipogenesis showed strong clustering and correlation of the lipid species. Panel (**A**) illustrates the matrix of the analysis where each square indicates the Spearman’s rank correlation coefficient. Positive correlations between the variables are shown in red, while negative correlations are shown in blue. Correlation matrix enables the assignment of six lipid clusters. Species were clustered using Ward’s clustering algorithm. Panel (**B**) shows the changes of the average lipid concentration in the different clusters with time.

**Figure 6:**
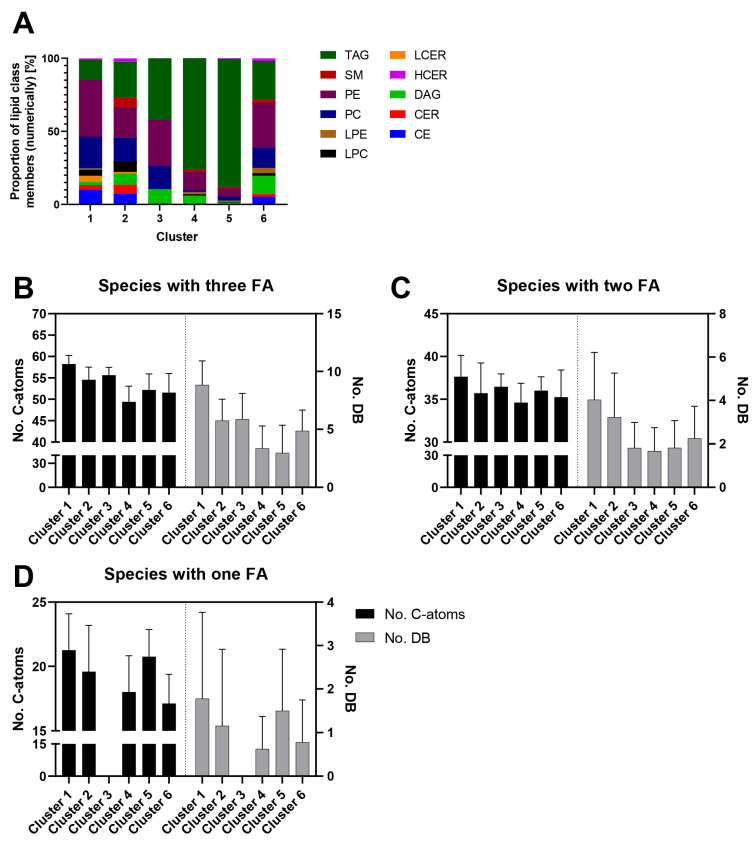
Clusters differed widely in their lipid class and lipid species composition. Panel (**A**) highlights the cluster compositions by showing the relative numerical proportions of the lipid class members. Panels (**B**–**D**) show the analysis of the FA side chain lengths and the number of DB based on the number of side chains of the lipid classes. Panel (**B**) shows the analysis of the class with three bound FA side chains, panel C that of lipid classes with two bound FA side chains, and panel D that of classes with only one FA side chain. Lipid species with patterns of decreasing concentration throughout adipogenesis had in general the highest numbers of DB and chain lengths, independent of their number of side chains.

The opposing trends in concentrations of some lipid classes raised the questionwhether they might be the result of an underlying regulatory network. To reveal possible associations between the lipid species, we computed pairwise Spearman’s rank correlations of lipid species concentration trajectories (Figure 5). The lipids could be assigned to six clusters (Figure 5A) and for each of the clusters the average concentration changes over time are displayed in Figure 5B. Species of different lipid classes were found to be distributed between the different clusters (Figure 6A and Figure S6).

Cluster 1 (*n* = 97 lipid species) consisted of lipid species with continuously decreasing concentration profiles (Figure 5B). In cluster 1, lipid species with the highest average FA side chain lengths (expressed as average total number of C-atoms) as well as number of double bonds (DB) were found, independent of the lipid class (Figure 6B–D). The cluster was dominated by PE (37.6%) and PC (20.8%) species. However, with the exception of SM, lipids from all other lipid classes were also clustered here (Figure 6A). Interestingly, 80% of all LCER species of the dataset could be found in this cluster (Figure S6A). In addition, more than 74.3% of the lipid species within this cluster had at least one FA side chain with at least 20 C-atoms and a high degree of desaturation (Table S5). Therefore, this cluster can be considered as the “PUFA cluster”.

Cluster 2 (*n* = 61) comprised species that exhibited decreasing concentrations starting at day 8. This cluster contained lipids of all classes except those of LPE. The species had approximately the third highest total number of C-atoms and DB (Figure 6B–D). Remarkably, cluster 2 contained a relatively high number of sphingolipids: 56.6% of all CER, 40.0% of all HCER, and 41.7% of all SM species of the dataset (Figure S6B) could be found in this cluster. Therefore, it can be characterized as the “sphingolipid and PUFA cluster”.

Cluster 3 species (*n* = 19) showed a fluctuating concentration course with a decrease in concentrations until day 8, followed by an increase to above the starting values. Four lipid classes were part of this cluster: TAG (42.1%), DAG (10.5%), PC (15.8%), and PE (31.6%). Remarkably, 15 out of 19 lipids (79%) contained at least one FA with 18 carbon atoms.

Lipids of clusters 4 and 5 (*n* = 198 and 312, respectively) were generally characterized by a strong concentration increase during adipogenesis. However, cluster 4 species increased substantially only until days 12–16 and decreased thereafter to half their maximum concentrations, while cluster 5 species reached a plateau at day 16. In cluster 4, TAG was the most abundant lipid class (75.8%), followed by PE (13.1%), and DAG (6.1%), while CE and all ceramide classes (CER, HCER, and LCER) were completely absent. The species of this cluster had the lowest number of C-atoms and DB within the dataset (Figure 6B–C). In cluster 5, the relative amount of TAG was even higher (88%) than in cluster 4 (Figure 6A). Owing to the high number of TAG species, clusters 4 and 5 also showed the highest lipid concentration values among all clusters (Figure 2B). Therefore, clusters 4 and 5 can be considered as “TAG clusters.”

The lipids in cluster 6 (*n* = 56) exhibited a fluctuating averaged concentration profile throughout the investigated time period. Lipid species of all classes except LCER were represented in this cluster, which was in general rather heterogeneous in terms of composition (Figure 6A).

The correlations between the clusters might reveal new insights into lipid remodeling. In general, we observed positive correlations between clusters 1 and 2 as well as between clusters 4 and 5. Remarkably, lipids from clusters 1 and 2 were strongly negatively correlated with lipids in clusters 4 and 5, which might be an indicator of lipid remodeling. Specifically, many TAG species from cluster 5 had strong negative correlations with more than a dozen PE species of cluster 1 (between −0.7 and −0.96). These PE species mostly contained polyunsaturated and VLCFA, whereas the TAG species were carrying at least one LCFA (Table S5). 

Moreover, some sphingomyelins, especially SM 20:0 (cluster 4), SM 22:0 (cluster 5), and SM 24:0 (cluster 5), had high negative Spearman’s correlation coefficients (down to −0.84) with species from classes CER, HCER, and LCER (all in clusters 1 and 2, Table S5). These results point to regulatory interactions between the lipid species over several lipid classes.

In addition, we also found high positive correlation coefficients (mostly between 0.85 and 0.98) between DAG and TAG species. Those DAG and TAG with very high co-correlations mostly contained one or two of the most abundant fatty acids (C16:0, 16:1, 18:0, and 18:1) as side chains. Additionally, these TAG were characterized by total C-atom numbers between 42 and 54, which further implies that LCFA were the main constituents. 

## 3. Discussion

In the present study we characterized the different stages of human adipogenesis by using the Lipidyzer™ method [26] which enabled the simultaneous quantification of 743 lipid species of 11 different lipid classes in differentiating human SGBS cells. This global lipid analysis enabled us to identify correlations between lipid species over several classes and opened the possibility to generate hypotheses on lipid remodeling during adipogenesis. 

Prior to the adipogenesis characterization, we analyzed the method performance in terms of linearity, repeatability, and background signals to be able to apply this novel methodology for accurate lipid analysis to SGBS cell culture samples. We used representative samples for undifferentiated and differentiated SGBS cells and validated the Lipidyzer™ method according to recently published studies that already showed good performance of the Lipidyzer™ method [21,22,23,24,25].

The lipid classes CE, CER, DAG, HCER, LCER, LPC, LPE, PC, PE, SM, and TAG could be analyzed with good linearities as indicated by coefficients of determination above 0.9 in at least one SGBS sample type. Furthermore, these lipid classes could be measured with high precision (CVs < 12%) which was determined by repeated analyses of QC samples from pooled SGBS homogenates. However, DCER and FFA had to be discarded from the data set, because the observed concentration levels were mostly in the range of the blank samples and therefore led to poor linearity and low precision. The detected high background signals of FFA in blank samples indicate high contaminations of these species that can be often found in glassware, pipette tips, and even in high-grade organic solvents [27]. We conclude that the Lipidyzer™ method was suited to reliably quantify 743 lipids from 11 lipid classes for the analysis of SGBS cell samples.

We are the first to use this novel method for the determination of lipid levels in differentiating human adipocytes. Additionally, we are also the first who characterized the human adipogenesis with this broad coverage of different lipid classes using only one method. Liaw and coworkers investigated the endpoints of adipogenesis (i.e., pre-adipocytes vs. fully differentiated adipocytes, day 12) in murine adipocytes of a large amount of lipids using an LC-MS/MS^ALL^ shotgun lipidomics approach [18]. They identified a shift from highly unsaturated VLCFA bound to the backbones of TAG, SM, cardiolipins, and ether-linked monoalkyldiacylglycerols in preadipocytes to more saturated LCFA in differentiated 3T3-L1 adipocytes. We could confirm these findings for VLCFA-carrying SM since we identified a decrease in their concentration levels until day 16. However, we measured an increase from day 16 to 20 almost reaching the concentration levels of day 0. The authors investigated adipogenesis at day 12, possibly missing the increase in the late phase of differentiation we observed. On the other hand, and different to the study of Liaw et al., we observed members of further lipid classes, namely CE, CER, DAG, HCER, LCER, LPC, PC, and PE species, having strongly decreased amounts of highly unsaturated VLCFA in fully differentiated adipocytes when compared to preadipocytes.

We followed the adipogenesis process over 20 days by analyzing samples from six time points (days 0, 4, 8, 12, 16, and 20). Successful cell differentiation of preadipocytes into lipid-laden adipocytes was confirmed on two different levels. First, we could observe the expected morphological changes during cell differentiation by microscopy. Second, the upregulation of *PPARG* and *CEBPA* expression showed strong transcriptional activation of cell differentiation. The very tight clustering of the data for samples from the same time point in the PCA and PLS-DA plots demonstrated generally high quality of the data obtained by the targeted lipidomics technique. Furthermore, the shift from differentiating (days 0–4) to maturating (days 4–12) SGBS cells became clearly visible by the change from PC1 to PC2 as the main contributor for cluster separation in the score plots. The observed shift coincides with findings from Halama and coworkers, who characterized murine adipogenesis of 3T3-L1 cells using a combined metabolomics and transcriptomics approach [16]. This shift during adipogenesis can be explained by the change from differentiation to maturation medium at day 4.

During ongoing adipogenesis we observed on the one hand strongly decreasing levels of CE, CER, HCER, and LCER, and on the other hand, substantially increasing levels of nearly all investigated glycerophospholipid classes, namely LPE, PC, PE, SM, as well as TAG. However, the temporal concentration courses of the individual lipid classes followed a particular pattern that was dependent on the developmental phases of the cells.

During the differentiation phase, we observed increasing concentrations of CER until day 4. The ceramides are known to be involved in signaling activity in cell cycle arrest and the inhibition of cell proliferation during early adipogenesis [28]. Both processes are required for the induction of cell differentiation of preadipocytes into adipocytes [29]. Thus, the time courses of CER concentrations give rise to the hypothesis that at least some of these compounds were involved during cell differentiation signaling. After day 4, when cell differentiation was completed and maturation started, we observed decreasing levels of CER together with HCER and LCER accompanied by a simultaneous increase of SM. This could be explained by the remodeling of all ceramides into SM. This is further supported by the fact that we observed high negative Spearman’s correlation coefficients between several SM species and CER, HCER, and LCER from the sphingolipid and PUFA cluster (cluster 2).

The differentiation phase was also characterized by decreasing concentration levels of CE. The CE operate as transport intermediates of cholesterol, which is an important component of the cell membrane [30,31]. Decreasing CE concentration levels indicate the release of cholesterol and its insertion into the cell membranes [32]. Incorporation of cholesterol decreases the flexibility of plasma membranes and thereby enables the morphological changes of the membranes that are important for differentiation and maturation. Furthermore, the cholesterol might also have been incorporated into triglyceride lipid droplet surfaces, serving as an intracellular free cholesterol reservoir [31]. These hypotheses are supported by negative correlations down to −0.78 between CE in the PUFA clusters 1 and 2 and TAG in the TAG clusters 4 and 5.

The second phase of adipogenesis, the maturation phase, was characterized by a strong increase of TAG from micromolar to millimolar levels. It is not surprising that TAG became the dominant lipid constituent of the adipocytes, as this reflects the function of adipocytes as sites for the storage and supply of fatty acids. The biosynthesis of TAG from DAG is corroborated by high positive correlations between these two lipid classes [33,34]. We observed a biphasic pattern of DAG and TAG concentration courses during adipogenesis. After a lag phase until day 4, considerable production of TAG via DAG reached a maximum at day 16 and then slowly declined. We conclude that the massive synthesis of TAG and their precursors started only after full differentiation from preadipocytes to adipocytes. As Collins and coworkers have shown by using ^13^C-labeled substrates, the massive generation of TAG is presumably based on de novo lipogenesis from glucose provided in the cell culture medium [19]. However, our correlation analysis revealed strong negative correlations between several PE species containing VLCFA and TAG. This might be an indicator of a possible contribution of PE containing VLCFA as an additional source for TAG synthesis during adipogenesis. The PE species might have been catabolized by phospholipase C to DAG, re-esterified to TAG, and incorporated into lipid droplets [35,36]. The increase of intracellular lipid depots is accompanied by expansion of the cell surface and volume, which requires larger amounts of the major membrane lipid classes like PC, PE, SM, and cholesterol [37,38,39]. Indeed, we observed simultaneous increases of PC, PE, and SM after day 4. Furthermore, LPC and LPE, which can be regarded as metabolic intermediates of PC and PE, also increased during adipogenesis [40].

Surprisingly, we observed the odd chain fatty acids C15:0 and C17:0 in CE, DAG, LPC, PC, PE, and TAG at non-negligible concentrations. Both fatty acids showed strongly increasing concentration time courses during adipogenesis. Roberts et al. also quantified increased odd chain fatty acid levels during cell differentiation of murine 3T3-L1 adipocytes [9]. We speculate that the increasing levels during adipogenesis might be explained by sequential peroxisomal fatty acid α-oxidation, which has been shown to occur in differentiating adipocytes [41].

Furthermore, some of the time courses of certain lipids might be explained by influences of the composition of the culture medium. To investigate a possible contribution of FA from the culture medium, we measured the FBS-containing medium for the cultivation of preadipocytes as well as the differentiation medium with the Lipidyzer™ method. Lipids with very long-chain PUFAs were highly concentrated in the FBS-supplemented medium compared with the levels in the differentiation medium lacking FBS (Figure S7). Therefore, we hypothesize that the measured concentration profiles of these lipid species during adipogenesis could have been artificially influenced by the cultivation with FBS-containing medium before the start of differentiation. As a result of this, the decreasing concentration levels of the VLCFA might also be explained by a lack of supply of these FA in the FBS-free differentiation and maturation media during adipogenesis.

It is important to keep in mind that cell culture experiments reflect artificial conditions. First, in vitro experiments often require high concentrations of growth factors, hormones, or several stimulation factors, which do not reflect physiological in vivo conditions. For instance, the differentiation of SGBS and other pre-adipocyte cells requires the corticosteroid dexamethasone and the insulin sensitizer rosiglitazone, which might strongly influence the lipidome. Indeed, Jeucken and Breuwens recently showed that rosiglitazone has an effect on the lipidome of HeLa cells [42]. In addition, the PPARγ agonist rosiglitazone as well as the endogenous myokine irisin can induce browning of white adipocytes [43]. Some recently published manuscripts showed this propensity of the SGBS cells towards a beige phenotype [43,44,45,46]. The used protocol for the SGBS cells requires an initial four-day stimulation with rosiglitazone for the induction of differentiation. This short time period might have an influence on the lipidome. However, undifferentiated SGBS cells behave very similar to human primary preadipocytes and the fully differentiated cells cannot be morphological distinguished from human primary adipocytes [47]. Moreover, one study compared SGBS cells, derived from subcutaneous adipose tissue of a male infant, with primary subcutaneous adipocytes from obese female patients [44]. The different confounders, obesity and sex, might also have a significant influence on the differentiation capacity and therefore also on the comparison of the two cell models. In addition, SGBS cells carry an FTO risk allele and the cells do not have a Simpson-Golabi-Behmel syndrome typical mutation in the glypican-3-gene (GPC3) what the name of the cell strain would suggest [47]. Nevertheless, future efforts will be necessary to confirm our findings in human primary subcutaneous adipocytes. Second, the lipid synthesis in adipose tissue *in vivo* is based not only on de novo synthesis from mostly glucose but also on circulating fatty acids in the bloodstream [48]. To sum up, cell culture experiments are indeed helpful to shed light on several cellular processes separately. However, owing to their simplicity and artificial nutrition, they cannot represent physiological in vivo conditions.

It also has to be mentioned that the Lipidyzer™ technology has some limitations. First, it was developed for the analysis of human plasma, which of course has a different lipid composition from (pre)adipocytes. The internal standard concentrations were therefore optimized for human plasma and may not match the actual situation in cell culture samples. Another issue concerns quantification. Although the Lipidyzer™ uses up to 10 internal standards (IS) per lipid class, there is no IS for each individual analyte. In addition, the method does not use a calibration curve for absolute quantification. Therefore, the measured absolute concentrations should be interpreted carefully. Furthermore, the Lipidyzer™ method is capable to determine the lipid species at the fatty acyl/alkyl level, except for the class of TAG. In case of TAG, the method cannot distinguish the *sn-1*, *sn-2*, and *sn-3* positions of the glycerol backbone and can as well not determine the exact positions of double bonds in the side chains. Besides, as the Lipidyzer™ method is a commercial assay with specialized and standardized software it is not possible to include other lipids into the method. This unfortunately limits the availability of further interesting lipid species such as for example signaling phospholipids.

Despite these limitations, we think that targeted analytical methods using multiple internal standards per lipid class—like the Lipidyzer™ technology—should be the methods of choice for the quantitative analysis of longitudinal samples with strongly different analyte concentrations and matrix conditions between sampling points. It has recently been shown by Chamberlain et al. that “due to the presence of matrix effects in untargeted, non-quantitative metabolomics, the signal intensity of any single analyte cannot be directly compared to the signal intensity of that same analyte (or any other analyte) between any two different matrices” [49]. This is of particular importance for ESI-MS-based lipid analytics, because matrix effects can vary considerably between lipid classes and even lipid species of the same class can respond differently to matrix effects depending on acyl chain length and degree of unsaturation [50,51]. Chamberlain et al. further concluded that “due to differences in ionization efficiency, the signal intensity of any single analyte cannot be directly compared to the signal intensity of any other analyte, even in the same matrix.” Thus, any kind of correlation or network analysis would be hampered with non-targeted or shotgun approaches. The application of IS can avoid or at least reduce the negative impact of matrix effects on the results. Non-targeted metabolomics or shotgun lipidomics approaches not including IS are more susceptible to matrix influences and should be therefore interpreted very carefully.

## 4. Materials and Methods 

### 4.1. Cell Culture, Cell Harvesting and Homogenization

The Simpson Golabi Behmel syndrome (SGBS) preadipocyte cell strain was provided by Martin Wabitsch. The cells were cultivated and differentiated for 20 days, as described previously [52]. In brief, 50,000 preadipocytes per well were seeded in six-well plates in DMEM/F-12 medium (Thermo Fisher Scientific, Waltham, MA, USA), supplemented with 10% FBS (Biochrom, Berlin, Germany), 3.3 mM biotin (Merck, Darmstadt, Germany), and 1.7 mM pantothenate (Merck, Darmstadt, Germany) and grown at 37 °C and 5% CO_2_ in a humidified atmosphere. Cell differentiation was initiated when cells reached about 90% confluence. At that point, the medium was exchanged for serum-free medium supplemented with 10 µg/mL transferrin (Merck, Darmstadt, Germany), 0.2 nM triiodothyronine (T3; Merck, Darmstadt, Germany), 250 nM hydrocortisone (Merck, Darmstadt, Germany), 20 nM human insulin (Merck, Darmstadt, Germany), 25 nM dexamethasone (Merck, Darmstadt, Germany), 250 µM 3-isobutyl-1-methylxanthine (IBMX; Merck, Darmstadt, Germany), and 2 µM rosiglitazone (Biomol, Hamburg, Germany). After 4 days and then every fourth day, thereafter, the medium was replaced with serum-free medium containing 10 µg/mL transferrin, 0.2 nM T3, 250 nM hydrocortisone, and 20 nM human insulin (maturation medium). Cell morphology was monitored by microscopy.

Cell samples for quantitative real-time PCR (qRT-PCR) analyses were taken in quadruplicates (biological replicates) at each of 5 time points beginning with the start of differentiation (representing day 0), then on day 2, 4, 8, and 12 of adipogenesis. Cell samples were processed as described below. Cell samples for Lipidyzer™ analyses were taken at six time points beginning with the start of differentiation (representing day 0) and then on every fourth day of differentiation until day 20. At each time point, six samples (biological replicates) were collected. Harvesting, homogenization of cells, and normalization of measured metabolite concentrations to the cell number were performed as recently reported [53], with minor modifications. In brief, after one washing step with 6 mL of warm PBS per well of a six-well plate, the cells were scraped off the wells in 500 µL of extraction solvent of ice-cold 80% methanol per well using rubber-tipped cell scrapers (Sarstedt, Nümbrecht, Germany). Harvested cell-solvent suspensions of four wells were pooled into pre-cooled 2 mL microtubes (Sarstedt, Nümbrecht, Germany) containing 400 mg of glass beads (Bertin, Frankfurt, Germany). The samples were stored at −80 °C until further use. Homogenization of cells was performed immediately before analyses at 4–10 °C twice for 25 s at 5500 rpm using a Precellys24 (PeqLab, Erlangen, Germany). The resulting homogenates were used for lipidomics measurement by FIA-(DMS)-MS/MS as well as for DNA quantification (DNA content indirectly reflects the cell number of the sample and was determined for normalization purposes) [53].

### 4.2. RNA Isolation and Quantitative Real-Time PCR (qRT-PCR)

Total RNA of four independent biological replicates per group was extracted from cells using miRNeasy mini kit (Qiagen, Hilden, Germany) according to the manufacturer’s protocol. Synthesis of cDNA was performed by using the RevertAid First Strand cDNA Synthesis Kit (ThermoFisher Scientific, Dreieich, Germany) according to the manufacturer’s instructions. Total RNA was reverse transcribed using an anchored oligo(dT)_18_ primer (5’-TTTTTTTTTTTTTTTTTTVN-3’) in a final concentration of 0.5 µM for priming cDNA synthesis. For qRT-PCR, primers were designed using Primer3 to span at least one exon-intron boundary to avoid falsified amplification results [54]. Primers were synthesized by Metabion (Planegg, Germany) and sequences were as follows: PPARG_for (5’-GACCACTCCCACTCCTTTGA-3’), PPARG_rev (5’-GAGATGCAGGCTCCACTTTG-3’), CEBPA_for (5’-AACAGCTGAGCCGCGAACTG-3’), CEBPA_rev (5’-CGGAATCTCCTAGTCCTGGCT-3’), TBP_for (5’-CAGCCGTTCAGCAGTCAA-3’), TBP_rev (5’-CTGCGGTACAATCCCAGAAC-3’). The amplification was performed on a QuantStudio Flex 7 Real-time PCR system (ThermoFisher Scientific, Dreieich, Germany) in triplicates using Power SYBR Green PCR Mastermix with ROX as passive reference (ThermoFisher Scientific, Dreieich, Germany) as follows: Denaturation at 95 °C for 10 min, 39 amplification and quantification cycles with 95 °C for 15 s and 60 °C for 1 min, and finally a melting curve program (95 °C for 15 s, followed by 60–95 °C with a heating rate of 0.1 °C/s) and continuous fluorescence measurement. The cycle threshold (CT) values were determined using the QuantStudio Flex 7 Real-time PCR system software. Relative gene expression was calculated using the comparative 2^−ΔΔCT^ method [55]. Amplification efficiencies were determined based on the slope of the calibration curve consisting of five different cDNA concentrations each measured in triplicates and efficiencies were as follows: *PPARG* (106.3%), *CEBPA* (96.3%), and *TBP* (88.7%). The fold-change values for gene expression were normalized by the respective efficiencies using a published procedure [55]. Relative gene expression data for *PPARG* and *CEBPA* were subsequently normalized to the reference gene of tata-box binding protein (TBP; in pre-experiments tested to be suited) and the gene expression of samples at day 0 of adipogenesis.

### 4.3. Hoechst Assay for DNA Quantification

For DNA quantification, the fluorochrome Hoechst 33342 (ThermoFisher Scientific, Waltham, MA, USA) was diluted in PBS to a final concentration of 20 µg/mL. A total of 80 µL of this solution was pipetted into each of the wells of a black 96-well plate (F96; Nunc, Thermo Fisher, Schwerte, Germany). Then, 20 µL of vortexed cell homogenates or plain solvent (80% MeOH; blanks) was added to the Hoechst solution and mixed by pipetting. The plate was incubated in the dark for 30 min at room temperature. Fluorescence signals were read using a GloMax Multi Detection System (Promega, Mannheim, Germany), equipped with a UV filter (λ_ex_. = 365 nm; λ_em._ = 410–460 nm, Promega, Mannheim, Germany) [53].

### 4.4. Lipid Extraction and Targeted Lipidomics Analysis

The Lipidyzer™ method (SCIEX, Darmstadt, Germany) was used to analyze the cellular lipidome. It detects lipids with fatty acid side chains of medium-chain (MCFA; C12), long-chain (LCFA; C13–C21), and very long-chain (VLCFA; C22–C26) lengths from 13 classes of lipids including cholesterol esters (CE), ceramides (CER), dihydroceramides (DCER), diacylglycerols (DAG), free fatty acids (FFA), hexosylceramides (HCER), lactosylceramides (LCER), lysophosphatidylcholines (LPC), lysophosphatidylethanolamines (LPE), phosphatidylcholines (PC), phosphatidylethanolamines (PE), sphingomyelins (SM), and triacylglycerols (TAG). The method allows the identification of lipid species at the fatty acyl/alkyl level (exception: TAG) [56]. The Lipidyzer™ method determines the sum of the numbers of C-atoms and double bonds (DB) for one fatty acid side chain as well as the sum of the C-atoms and DB of all three side chains. The notation rules from Liebisch and coworkers only know the case that either no fatty acid is known (e.g., TAG 52:2) or all three (e.g., TAG 16:0_18:1_18:1) [56]. Therefore, the nomenclature for TAG species in our study was adopted to these recommendations. The internal standard (IS) mixture (Avanti Polar Lipids, Inc., AL, USA) was prepared in accordance to the Lipidyzer™ manual. For QC samples, 250 µL of pooled cell homogenates were used, consisting in equal parts of undifferentiated, differentiating (day 8 of differentiation), and maturely differentiated cells (day 16). Three reference plasma samples (SCIEX, Darmstadt, Germany) of 100 µL in volume were spiked each with 50 µL of the QC spike mixture (SCIEX, Darmstadt, Germany) to investigate inter-run and inter-project effects. Lipids were extracted by two-phase separation using methyl *tert*-butyl ether (MTBE), methanol, and water [57]. Briefly, 250 µL of cell homogenates for the main experiments or 10–300 µL for method evaluation experiments, QC samples, or QC spiked plasma samples were transferred to 1.5 mL safe-lock reaction tubes (Eppendorf, Hamburg, Germany). For each time point, we took in total six biological independent cell samples. Next, 160 µL of MeOH and 900 µL of MTBE were added to each tube and incubated for 30 min at 900 rpm and room temperature in a shaker. For phase separation, 500 µL of H_2_O was added to each tube, the mixtures were vortexed, and the tubes were centrifuged at 15,000× *g* for 4 min at RT. The upper organic phases were transferred into glass vials. The extraction step was repeated once and organic phases were combined. Organic solvents were evaporated to complete dryness under a stream of gaseous nitrogen and residuals were reconstituted with 250 µL of sample running buffer (10 mM ammonium acetate in dichloromethane:methanol (50:50 *v*/*v*)). Samples were then analyzed with the Lipidyzer™ method, consisting of a Sciex 5500 MS/MS QTRAP system (SCIEX, Darmstadt, Germany) equipped with a SelexION ion source for differential mobility spectrometry (DMS), in accordance with the manufacturer’s instructions [24]. A sample volume of 50 µL was injected with a Shimadzu Nexera X2 liquid chromatography system (SCIEX, Darmstadt, Germany) at an isocratic flow rate of 7 µL/min. Data were acquired automatically with the Lipidyzer™ Workflow Manager software (version 1.0, SCIEX, Darmstadt, Germany). The obtained concentration values in nmol/g supplied by the software were converted to µmol/L with the assumption that 1 mL of cell culture sample was equal to 1 mL of plasma which is equal to 1 g [58]. Converted concentration values were normalized by Hoechst assay results. 

### 4.5. Data Analysis

To trace the process of adipogenesis, the concentrations of the single lipid species, summed concentrations of lipid classes, concentrations of fatty acids, and percentage compositions were analyzed. Lipid species were completely excluded from the data set if concentration values were missing (NA) in more than 33.3% of the samples within a time point. Missing values were replaced by the respective minimal lipid species concentration measured, divided by √2 and multiplied by a randomly chosen factor between 0.75 and 1.25.

Statistical analyses and graphical illustrations of the lipidomic data were performed using the software MetaboAnalyst 4.0 [59], GraphPad Prism 8.1.1, and R 3.5.1 [60].

Univariate statistical analyses were performed using the Mann–Whitney U test and Kruskal–Wallis test with Dunn’s post-hoc test. Spearman’s rank correlation analysis was used to test correlations between lipid species. Prior to PCA and other multivariate statistical analyses, lipid concentrations were log-normalized and auto-scaled (mean-centered and divided by the standard deviation of each variable) to achieve a normal distribution of the data set. Averaged concentrations are shown with standard deviations. 

## 5. Conclusions

We used the Lipidyzer™ technology to study the cellular lipidome during the development of preadipocytes into maturating and finally mature adipocytes in a human cell culture model. The switch from differentiating preadipocytes to maturating adipocytes became clearly visible at the lipidome level. The differentiation process was accompanied by increased concentrations of ceramides that are known to be involved in cell differentiation signaling. While these ceramide species decreased after completion of differentiation around day 4, massive lipid remodeling occurred during maturation of the adipocytes. This maturating phase was characterized by the substantial synthesis of DAG and TAG species. We furthermore observed increases of membrane lipids like PC, PE, and SM as well as their biosynthetic precursors. Moreover, we could also show that the compositions of the lipid species itself became more homogeneous during differentiation to highly concentrated saturated/monounsaturated LCFA with the four most abundant fatty acids being C16:0, C16:1, C18:0, and C18:1. Interestingly, VLCFA constantly decreased in almost all investigated lipid classes during the maturation process. High negative correlation coefficients between membrane lipids containing VLCFA and TAG species imply that these lipids might have served as additional sources for TAG synthesis. However, further studies are necessary to shed light on other lipid classes, lipid synthesis, degradation, and remodeling pathways during adipogenesis. For instance, fluxome-based approaches could be helpful to follow especially polyunsaturated and VLCFA within the preadipocytes. Moreover, a multi-omics approach might be helpful to detect connections between different pathways within the lipidome as well as the connection with pathways of more polar metabolites. In addition, it would also be interesting to compare the lipidomes of SGBS cells and freshly isolated human adipocytes since the metabolism in these cells might better reflect the original situation in humans. We could also show that the cultivation of cells with FBS-containing medium might influence the metabolism of the cells from several days up to weeks later. We recommend analyzing the medium used for cell culture lipidomics/metabolomics studies to prevent misinterpretation of the data.

## Supplementary Materials

The following are available online at https://www.mdpi.com/2218-1989/10/6/217/s1, Figure S1: Analytical evaluation of the Lipidyzer™ method for undifferentiated and differentiated cells revealed strong linearity for most of the analyzed lipid classes; Figure S2: Representative microscopic images of SGBS cells showed successful differentiation based on morphological changes and a strong increase in number and size of lipid droplets; Figure S3: Transcripts of the main adipogenic transcription factors PPARγ (*PPARG*) and C/EBPα (*CEBPA*) were highly upregulated during adipogenesis; Figure S4: Principal component analysis (PCA) score plot showing very clear clustering of lipid species regarding the different time points of adipogenesis; Figure S5: Fatty acid concentrations and compositions changed markedly during adipogenesis in all 11 lipid classes; Figure S6: Clusters from the spearman’s rank correlation analysis consisted of distinct lipid class compositions; Figure S7: Polyunsaturated and very long-chain fatty acids were highly concentrated in FBS-containing medium used for cultivation before differentiation start; Table S1: Linearity testing of method; Table S2: Repeatability and background signal testing; Table S3: Top 30 variable importance in projection (VIP) of PLS-DA for component 1 and 2, respectively; Table S4: Significances of Kruskal Wallis test; Table S5: Spearman’s rank correlation coefficients.
